# Genome-wide characterization and expression analysis of MYB transcription factors in *Gossypium hirsutum*

**DOI:** 10.1186/s12863-016-0436-8

**Published:** 2016-09-09

**Authors:** Haron Salih, Wenfang Gong, Shoupu He, Gaofei Sun, Junling Sun, Xiongming Du

**Affiliations:** 1 State Key Laboratory of Cotton Biology/Institute of Cotton Research, Chinese Academy of Agricultural Science (ICR, CAAS), Anyang, 455000 China; 2 College of Life Sciences, Huazhong Agricultural University, Wuhan, 430070 Hubei China; 3 Department of Computer Science and Information Engineering, Anyang Institute of Technology, Anyang, China; 4 Zalingei University, Central Darfur, Sudan

**Keywords:** Comparative genomics analysis, Upland cotton, MYB genes, Fiber development

## Abstract

**Background:**

MYB family proteins are one of the most abundant transcription factors in the cotton plant and play diverse roles in cotton growth and evolution. Previously, few studies have been conducted in upland cotton, *Gossypium hirsutum*. The recent release of the *G. hirsutum* genome sequence provides a great opportunity to identify and characterize the entire upland cotton MYB protein family.

**Results:**

In this study, we undertook a comprehensive genome-wide characterization and expression analysis of the MYB transcription factor family during cotton fiber development. A total of 524 non-redundant cotton MYB genes, among 1986 MYB and MYB-related putative proteins, were identified and classified into four subfamilies including 1R-MYB, 2R-MYB, 3R-MYB, and 4R-MYB. Based on phylogenetic tree analysis, MYB transcription factors were divided into 16 subgroups. The results showed that the majority (69.1 %) of *GhMYBs* genes belong to the 2R-MYB subfamily in upland cotton.

**Conclusion:**

Our comparative genomics analysis has provided novel insights into the roles of MYB transcription factors in cotton fiber development. These results provide the basis for a greater understanding of MYB regulatory networks and to develop new approaches to improve cotton fiber development.

**Electronic supplementary material:**

The online version of this article (doi:10.1186/s12863-016-0436-8) contains supplementary material, which is available to authorized users.

## Background

Plant growth and development are controlled by multigene families. Transcription factors play a key role in the regulation of gene transcription and commonly comprise four distinct domains: a DNA-binding domain, a nuclear localization signal, a transcription activation domain, and an oligomerization site [1]. These four domains work together to control many aspects of plant growth and development by activating or suppressing the transcriptional process [2]. Additionally, transcription factors are regularly encoded by multigene families which makes analyzing their individual roles more complex [1]. Compared with fungi and animals, the MYB transcription factors of higher plants are more broadly dispersed in the genome [3]. The MYB domain is highly conserved among plants, and proteins usually contain between one and four repeats (SONT domains) named R1, R2, R3, and R4. Each repeat is comprised of 50–53 amino acids which encode three α-helices, the second and third of which form a helix–turn–helix (HTH) structure [4]. The third α-helix forms the transcription factor DNA recognition site and interacts with the major groove of DNA [5]. Moreover, it is comprised of regularly spread triplet tryptophan residues that group together to make a hydrophobic core [6]. In contrast, the C-terminal promoter domain of different MYB proteins is quite diverse, leading to the broad variety of regulatory roles of the MYB gene family [7, 8]. The MYB domain was first identified in the avian myeloblastosis virus (v-myb) [9] and three additional MYB genes (c-myb, A-myb, and B-myb) were identified in different organisms such as vertebrates, insects, fungi, and slime molds [7, 10, 11]. The corn C1 gene was the first MYB gene identified in plants, and encodes a c-myb-like transcription factor responsible for the regulation of anthocyanin biosynthesis [12]. Generally, R2R3-MYB domain proteins are the predominant form found in higher plants [8].

To date, the plant transcription factor database (http://planttfdb.cbi.edu.cn/) contains approximately 8746 MYB, and 6410 MYB-related, sequences [13]. These genes may be involved in various plant cell activities such as secondary metabolism, hormone signaling [8, 14], environmental stress, cell development [15], and organ growth [16, 17].

Recently, several genome-wide analyses of MYB transcription factors have been conducted in *Arabidopsis*, rice [18], maize [19], *Salvia miltiorrhiza* [20], soybean [21], apple [22], sugarcane [15], and Chinese cabbage [23]. These studies can be utilized to identify MYB transcription factors in other plants, including cotton. However, very little information about MYB gene diversity and abundance in upland cotton is available. Several studies have shown that the MYB transcription factor family has a role in regulating fiber progress in cotton. The *GhMYB*109 and *GhMYB*2 genes, that belong to the R2R3-MYB subfamily, are associated with positive regulation of cotton fiber development [24, 25]. Moreover, silencing of *GhMYB*25 is involved in the production of short fibers in cotton [26], while suppression of *GhMYB25*-like produces fiber-less cotton [27]. The *GhCPC* gene belongs to the 3R-MYB subfamily and is a negative regulator of cotton fiber elongation [28]. A recent report has revealed that ten MYB (MIXTA-like) genes were highly expressed during early fiber development in *Gossypium hirsutum* (cultivar). However, examination of gene expression in three naked seed (fiber-less mutants) cotton mutants revealed that only one group of MIXTA-like genes had decreased expression levels [29].

Upland cotton is one of the most important fiber crops in the world and provides raw material for the textile industry [30]. Transcriptome analyses showed that several pathways were regulated in developing cotton fibers [31]. The status of those up-regulated or down-regulated pathways, and the molecular mechanisms by which they are controlled requires further investigation [32, 33]. As MYB proteins are one of the largest transcription factor families in higher plants, they may play key roles in regulating diverse pathways in cotton during fiber development [30]. Comprehensive analysis of upland cotton MYB proteins and their evolutionary variations, through tetraploid cotton, might help to reveal critical molecular mechanisms of cotton development and growth. In addition, the recent release of the *G. hirsutum* genome sequence [34] provides a great tool to identify and characterize the entire MYB protein family in upland cotton.

Here, we conducted a comprehensive genome-wide analysis of the MYB transcription factor family in *G. hirsutum*. A total of 524 MYB transcription factor encoding genes were identified and subsequently subjected to systematic analyses including: phylogenetic tree analysis, chromosomal location determination, gene structure analysis, conserved motifs identification, transcriptome (RNA-seq) analysis, and qRT-PCR analysis of selected MYB genes. Our genome-wide analysis of the *GhMYB* gene family might contribute to future studies on the functional characterization of MYB proteins in *G. hirsutum*. These research findings will provide information fundamental to determining the molecular and regulatory mechanisms of MYB transcription factors in cotton.

## Methods

### Identification of MYB gene family in upland cotton

Upland cotton protein sequences were downloaded from the Cotton Genome Project (http://cgp.genomics.org.cn/page/species/download.jsp?category=hirsutum) for computational analysis. Corresponding protein sequences were downloaded from the *Arabidopsis* database (TAIR; http://www.Arabidopsis.org/), cacao, mays, *Vitis vinifera*, *Populus trichocarpa* and *Gossypium raimondii* sequences were downloaded from the plant transcription factor database (http://planttfdb.cbi.edu.cn/). A local BLASTP search was performed to identify candidate MYB members, using *Arabidopsis*, cacao, mays, *Vitis vinifera*, *Populus trichocarpa* and *G. raimondii* MYB protein sequences as the query. Hits with e-values of 1e- 10 were deemed to be members of the MYB family. Furthermore, to confirm the protein sequences derived from the selected cotton MYB, candidate genes were examined using the domain analysis programs of Pfam (Protein family: http://pfam.sanger.ac.uk/) and SMART (Simple Modular Architecture Research Tool: http://smart.embl-heidelberg.de/). All redundant sequences were manually discarded, resulting in 524 MYB protein sequences. Additional analysis was based on cluster W alignment results.

### Mapping MYB genes on chromosomes

The chromosomal position of all *GhMYB* genes was determined through BLASTN searches against the *G. hirsutum* genome project (Cotton Genome Project of the Institute of Cotton Research of Chinese Academy of Agricultural Sciences). *GhMYB* genes were mapped on the chromosome using the Map Chart software. Two types of gene duplications were identified: tandem and segment duplication events. Gene duplications were identified provided that the length of the aligned sequence covered >80 % of the longer gene, and that basic on the similarity of the gene alignment regions was >80 % [35]. In addition, to further estimate *GhMYB* genes duplication events, the synonymous (Ks) and non-synonymous (Ka) substitution rates of evolution were calculated using the DnaSP software (version 5.10) [36]. Ka/Ks calculator was run on those *GhMYB* gene pairs to estimate their synonymous and non-synonymous rates of evolution. To estimate the evolutionary time of duplicated genes, Ks values were translated into duplication time in millions of years based on a rate of 1 substitution per synonymous site per year. The duplication events time (T) was calculated as T = Ks/2λ × 10^−6^ Mya (approximate value for clock-like rate λ = 1.5 × 10^−8^ years for cotton) [37].

### Phylogenetic analysis

The phylogenetic tree of MYB transcription factor genes was generated using multiple sequence alignments of upland cotton, *Arabidopsis* and cacao MYB protein sequences using Cluster W (http://www.ebi.ac.uk/Tools/msa/clustalw2/). Phylogenetic and molecular evolutionary analyses were performed using MEGA 6.0 software (http://www.megasoftware.net) with pairwise distance and the Neighbor-Joining (NJ) method. The tree was constructed with the following parameters: Substitution, Poisson Model; data subset to use, the p-distance, complete deletion; replication, bootstrap analysis with 1,000 replicates. Moreover, maximum likelihood and minimum evolution methods were also used in our phylogenetic tree to validate the result from the NJ method. Additionally, a separate phylogenetic tree was constructed with all the *GhMYB* protein sequences in *G. hirsutum* for further analysis.

### Gene structure analysis and identified motifs

MYB genomic and cDNA sequences were obtained from the Cotton Genome Project of the Institute of Cotton Research of Chinese Academy of Agricultural Sciences (http://cgp.genomics.org.cn/page/species/download.jsp?Category=hirsutum). The Online Gene Structure Display Server (GSDS 2.0) (http://gsds.cbi.pku.edu.cn/index.php) was used to examine gene structure by comparing each cDNA sequence with the corresponding genomic sequence. Conserved protein motifs in *G. hirsutum* MYBs were identified using the MEME program (version 4.8.2) (http://meme.nbcr.net/meme/intro.html). The following parameters were used: any number of repetitions, the maximum number of motifs-20, and optimum width from 6 to 250.

### Plant materials, RNA extraction and qRT-PCR analysis

Upland cotton (*Gossypium hirsutum L*.) Ligon-lintless 1 (Li1) mutants and wild-types (TM-1) seeds were provided from Institute of Cotton Research, Chinese Academy of Agricultural Sciences (CAAS, Anyang, China) and planted in the experimental field at the Institute of Cotton Research under conventional field management conditions. Flowers on one day before anthesis were tagged for self-pollination. To detect the MYB gene expression, samples were collected from ligon-lintless1 and wild-type cotton at different stages of cotton fiber development: 0, 3, 5, 8, 10, and 15 DPA. RNA was extracted from cotton ovules and fibers using the RNA Aprep Pure Plant Kit (Tiangen). The quality and concentration of each RNA sample was determined using gel electrophoresis and a NanoDrop 2000 spectrophotometer (Only that met the criterion 260/280 ratio of 1.8-2.1, 260/230 ratio ≥ 2.0) were used for further analyses and stored at −80 °C. High quality RNA samples were treated with DNase I (TaKaRa, Japan) to eliminate contaminating genomic DNA. The cDNA was synthesized from 2 μg of RNA in a 20 reaction volume using ReverTra Ace qPCR RT kit (TOYOBO, Japan) according to the manufacturer’s manual. qRT-PCR experiments were conducted to measure expression levels of MYB transcription factor family genes during cotton fiber development. qRT-PCR analysis was performed using the Applied Biosystems 7500 Real-Time PCR system and the SYBER premix ExTaq kit (TaKaRa. Japan). Target gene amplification was checked by SYBR Green fluorescence signal. The cotton constitutive β-actin gene was used as a reference gene and specific MYB primers were used for qRT-PCR. The following thermal cycle conditions were used: 95 °C for 2 min, followed by 40 cycles of 95 °C for 5 s, products collected at 60 °C for 34 s. All reactions were repeated three times with three biological replicates. Expression levels were calculated as the mean signal intensity across the three replicates. Following the PCR, a melting curve analysis was performed. Ct or threshold cycle was used for relative quantification of the input target number. Relative fold difference (N) is the number of treated target gene transcript copies relative to that of the untreated gene transcript copies, and is calculated according to Schmittgen et al. 2001 [38] as follows:

$$ \mathrm{N}={2}^{\varDelta \varDelta \mathrm{C}\mathrm{t}}={2}^{\left(\varDelta \mathrm{C}\mathrm{t}\ \mathrm{t}\mathrm{reated}\hbox{-} \varDelta \mathrm{C}\mathrm{t}\ \mathrm{control}\right)} $$

Where ∆∆Ct = ∆Ct of the treated sample minus ∆Ct of the untreated control sample, and ∆Ct is the difference in threshold cycles for *GhNAC18* target and the *GhActin1* internal reference.

### RNA-seq data analysis

To analyze upland cotton MYB expression patterns, we used Illumina RNA-seq data, including five stages of cotton fiber development (−1, 1, 3, 5, and 10 DPA) from wild-type (TM −1) upland cotton. Gene expression levels were calculated as reads per kilobase of exon model per million mapped reads (RPKM) units (Additional file 1: Table S1). Fold changes of different genes expression analysis and the related statistical computations of the two tested conditions were performed using the DESeq R package (1.10.1). The resulting *P*-values were adjusted using Benjamini’s and Hochberg’s method to control the false rate [39]. Only genes with an adjusted *P*-value < 0.05 found using DESeq were categorized as differentially expressed. Heat maps were generated and hierarchical clustering was performed using genesis_v1.7.6.30.09.10-DIGERATI software.

## Results and discussion

### Genome-wide identification of upland cotton *GhMYB* transcription factors

MYB transcription factor encoding genes of *G. hirsutum* and homologous MYB genes collected from *Arabidopsis,* cacao, mays, *Vitis vinifera*, *Populus trichocarpa* and *G. raimondii* were analyzed. Approximately 1986 MYB (MYB DNA-binding domain, contains ~52 amino acid residues in length and forms a helix-turn-helix fold with three regularly spaced tryptophan residues) and MYB-related (MYB-related genes are those transcription factors they have only one MYB domain. MYB domain proteins are more prevalent) [18] putative protein sequences were associated with the upland cotton genome. Of these, 582 non-redundant MYB sequences, which met the crucial value of 1 e- 10, were obtained. Furthermore, *GhMYB* candidate genes were examined using domain analysis programs of Pfam and SMART. A total of 524 non-redundant *GhMYB* genes were identified and considered for further analysis. These genes were classified into four distinct subfamilies including: 1R-MYB, 2R-MYB (R2R3-MYB), 3R-MYB (R1R2R3-MYB), and 4R-MYB (Additional files 2: Table S2 and Additional file 3: Figure S1) based on the number and location of MYB repeats. Our results indicate that, consistent with results observed in rice, *Arabidopsis* [18], Chinese cabbage [23], and apple [22], the majority of *GhMYB*s in upland cotton belong to the 2R-MYB sub-family (69.1 %). The second largest group 1R-MYB, accounted for 27.67 % of all *GhMYB* genes, while 3R-MYB and 4R-MYB accounted for 2.86 and 0.38 %, respectively (Table 1).

**Table 1:** The different subfamilies of *GhMYB* transcription factor types distributed on upland cotton chromosomes

Chromosome	Transcription factor Types				
	R2R3-MYB	R1-MYB	R1R2R3-MYB	Atypical MYB genes (4R-MYB)	TOTAL
At_chr1	10	3			13
At_chr2	6	3			9
At_chr3	3	0			3
At_chr4	8	2	1		11
At_chr5	6	3	1		10
At_chr6	11	7			18
At_chr7	18	6			24
At_chr8	6	1			7
At_chr9	19	9			28
At_chr10	5	2			7
At_chr11	6	2	1		9
At_chr12	11	3			14
At_chr13	17	1	1		19
At_Sub	126	42	4	0	172
Dt_chr1	17	4			21
Dt_chr2	4	2			6
Dt_chr3	6	2			8
Dt_chr4	7	1			8
Dt_chr5	14	4			18
Dt_chr6	14	4	2		20
Dt_chr7	9	5			14
Dt_chr8	14	4	1		19
Dt_chr9	17	12	1		30
Dt_chr10	9	3	2		14
Dt_chr11	8	2			10
Dt_chr12	6	2			8
Dt_chr13	21	3	1		25
Dt_Sub	146	48	7	0	201
scaffold	90	55	4	2	151
TOTAL	360	145	15	2	524
%	69.1	27.67	2.86	0.38	100

### Chromosomal distribution and annotation MYB genes

**Fig. 1:**
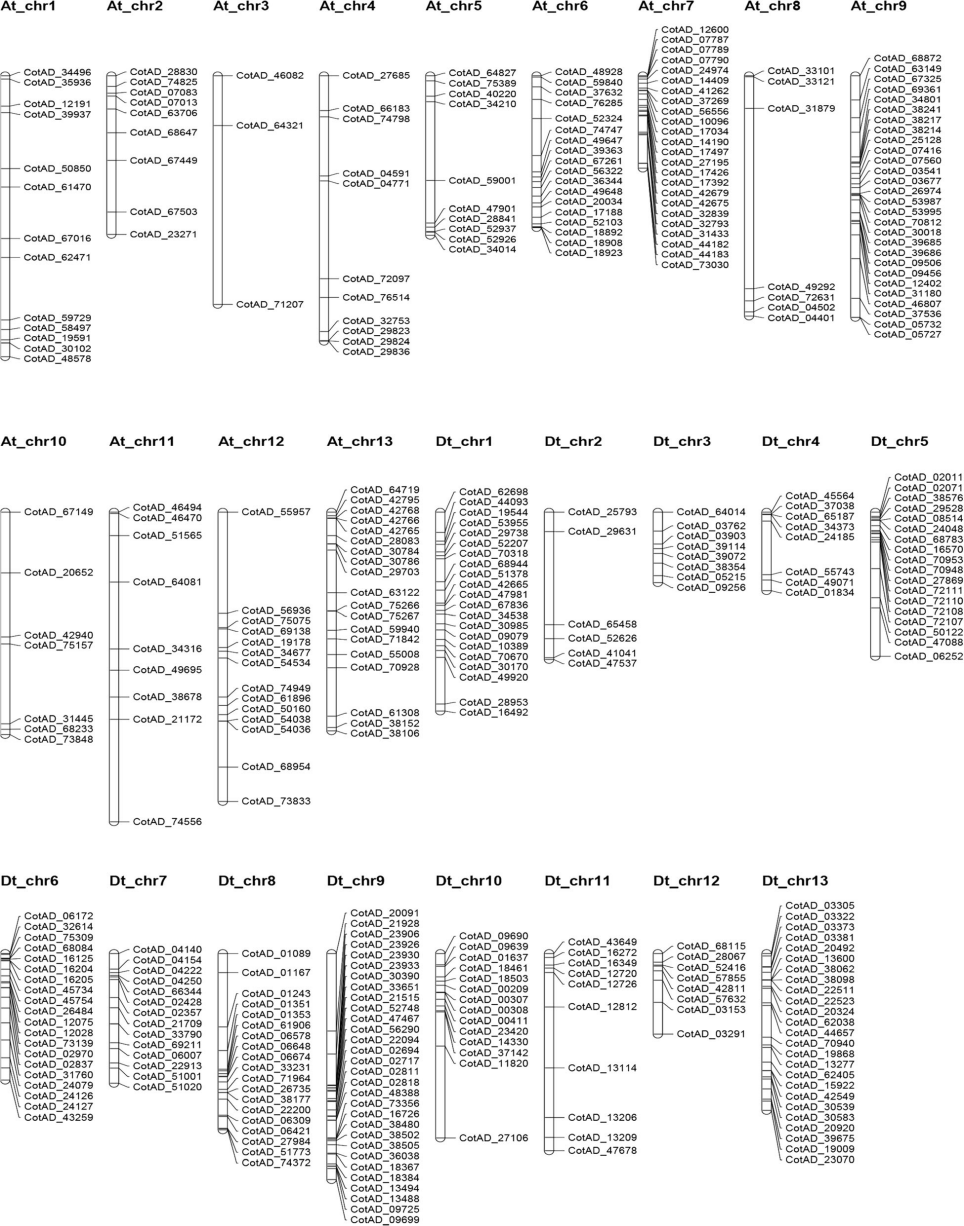
Distribution of *GhMYB* genes on cotton chromosomes. The chromosomal position of each *GhMYB* was mapped to the upland cotton genome

Analysis of the *G. hirsutum* genome sequence revealed 524 possible members of the *GhMYB* gene family. Of these genes, 114 had been annotated previously. Three hundred and seventy three (373) *GhMYB* transcription factor genes were mapped onto upland cotton chromosomes and named according to their chromosomal order (from chromosome 1 to 26) as *GhMYB*1 to *GhMYB*373. One hundred and fifty one (151) *GhMYB* genes were not obviously mapped to any chromosome (scaffolds), and named *GhMYB*374 to *GhMYB*524, respectively (Additional file 4: Table S3). The distribution and density of MYB transcription factor genes on chromosomes was not uniform. Some chromosomes, and chromosomal regions, have a high density of MYB transcription factor genes while others do not (Fig. 1). The highest density of MYB genes was observed on chromosome At 9 and its homolog chromosome Dt 9 (23) with 58 genes, and the lowest density of MYB genes was observed on chromosome At 3 and its homolog chromosome Dt 3 (17), with 11 genes. In addition, the majority of MYB transcription factor genes were found at the upper and centromeric regions of the chromosomes. In addition, a greater number of MYB genes were located on Dt chromosomes (tetraploid D) than on At chromosomes (tetraploid A) with 201 and 172 genes, respectively (Table 1).

Tandem and segmental duplication events are the main causes of gene-family expansion in upland cotton. Based on the whole genome analysis of gene duplications, 73 duplicated *GhMYB* gene pairs were made by segmental and tandem duplication, including 40 duplication events within the At and Dt chromosomes as well as 33 duplication events between the At, Dt chromosomes and scaffold (Additional file 5: Table S4), indicating that segmental duplications and tandem duplications contributed to the expansion of *GhMYB* in upland cotton*.* At list, two or more *GhMYB* genes reside on the same chromosome or on different chromosomes. A tandem duplication event is when gene duplication happens within the same chromosome while segmental duplication is when duplicated genes are located in different chromosomes. In this study, clusters formed by *GhMYB*s in the upland cotton (AD) genome were identified to explain the mechanism behind the expansion of the *GhMYB* family in cotton. We found that 5 gene pairs duplicated tandemly into chromosomes (At_chr5, At_chr7, At_chr12, Dt_chr5 and Dt_chr7) and 68 gene pairs duplicated segmentally, which deeply contributed to the expansion of the *GhMYB* transcription factors in upland cotton. The results also indicated that, among the duplication events in the *GhMYB* transcription factor family, the gene pairs that appeared to be derived from segmental duplication events occurred earlier than those that arose from tandem duplication (Additional file 5: Table S4). A gene duplication event, occurring during the course of cotton evolution, has led to the creation of new gene functions [40]. The origin of multigene families has been attributed to a region-specific gene duplication that occurred in upland cotton [34]. Furthermore, to calculate the evolutionary time of these identified MYB in *G. hirsutum,* an estimation of their synonymous and non-synonymous substitution rates during evolution, Ks and Ka values were calculated using the DnaSp software. Nucleotide substitutions in protein-coding genes can be categorized as synonymous or non-synonymous substitutions as elaborated in (Additional file 5: Table S4). The Ka/Ks ratio is a measure used to examine the mechanisms of gene duplication evolution after divergence from their ancestors. A Ka/Ks value of 1 suggests neutral selection, a Ka/Ks value of <1 suggests purifying selection, and a Ka/Ks value of >1 suggests positive selection (Hurst 2002). Here, we estimated synonymous and non-synonymous substitutions ratios (Ka/Ks) for the 73 pairs of segmentally and tandemly duplicated genes. It was found that most of *GhMYB* genes had Ka/Ks values of less than 1, implying that *GhMYB* genes have evolved under the effect of purifying selection while 17 duplicated genes had the Ka/Ks ratio more than 1, implying that those had evolved under positive selection. We further used Ks to estimate the time of *GhMYB* genes duplication events during the evolutionary time of upland cotton genome. The tandem and segmental duplication events in upland cotton that occurred between 0.26 (Ks = 0.0078) and 124.42 mya (Ks = 3.7326), with an average of 46.494 mya (million years ago). The Ks of tandem duplications of *GhMYB* genes occurred from 2.76 (Ks = 0.0828) mya to 44.343 (Ks = 1.3303) mya, with average 26.0493 mya. The results suggest that the expansion of the *GhMYB* genes in upland cotton which originated from At and Dt genomes mostly arose from whole genome duplication events during their evolution.

### Phylogenetic analysis of MYB transcription factors genes in upland cotton

To identify potential relationships between the various *GhMYB* gene family members, a Neighbor-Joining (NJ) phylogenetic tree was constructed. Examination of protein sequence similarity and phylogenetic tree analysis allowed us to divide the 524 upland cotton MYB genes into 16 subgroups, which ranged in size from 2 to 68 MYB genes (Additional file 6: Figure S2A). The bootstrap values for some subgroups of the NJ tree were low as a result of relatively large number of gene sequences that were also found in earlier study [23]. Supporting the phylogenetic analysis of subgroups, most *GhMYB* protein domain repeats exhibited high similarities within subgroups. Hence, we strongly sought other evidence to check the reliability of our phylogenetic tree. The phylogenetic trees of MYB TFs were reconstructed with maximum likelihood and minimum evolution methods to validate the result from the NJ and pairwise distance method. The trees constructed by the three methods mentioned above, are almost the same with only minimal differences at some subgroups (subgroup 7 and 16), implying that the tree methods were mainly consistent with each other (Additional file 7: Figure S3).

### MYB gene structure analysis and conserved motif identification

Gene structure analysis of 524 *GhMYB* transcription factors was performed. To provide greater insight into their intron/exon structure, cDNA and corresponding genomic sequences were compared. Approximately 90.84 % of upland cotton *GhMYB* genes contained between 1 and 12 introns. Similar to that described in *Arabidopsis*, *Vitis vinifera*, and *Eucalyptus grandis* [41, 42], most *GhMYB* transcription factors contained 1 (17.4 %) or 2 introns (52.7 %). The remaining 22.51 % of *GhMYB* genes contained more than two introns. However, only 8.2 % of *GhMYB* transcription factor genes contained no introns. Most of the intronless genes were clustered into the 13 and 21 subgroups. Furthermore, an uprooted phylogenetic tree was constructed using *GhMYB* protein sequences to assess the similarities in intron/exon structure within *GhMYB* gene subgroups (Additional file 6: Figure S2A). Within subgroups the majority of *GhMYB* genes contained similar exon/intron distribution arrangements, particularly related to exon length and intron number. Most of the 2R-MYB transcription factor genes had a conserved gene structure with three exons and two introns. Additionally, the size of the third exon was more variable than that of the first and second exon (Additional file 6: Figure S2B). High levels of variation in the sequence of the third exon are reported to be associated with functional divergence among R2R3-MYB genes [43]. Whilst few R2R3-MYB genes contained no intron, they were clustered into 13 subgroups. It was noted that the duplication of 2R during the early development of MYB proteins containing two repeats gave rise to the 3R-MYB domains. Therefore, it was found that most of the 1R-MYB, 3R-MYB, and 4R-MYB genes were disrupted by more than four introns. This is consistent with previous reports which have suggested that most MYB-related genes in higher plants contained more than 2 introns [44]. Within each subgroup, most of the *GhMYB* genes generally had similar intron/exon structures, as it was described previously by Jiang, Gu, and Peterson and Matus [42, 45]. There was a strong connection between the phylogeny tree analysis and the intron/exon structure of the *GhMYB* transcription factor family in upland cotton.

Further investigation of the variation within the conserved motifs of *GhMYB* proteins using the MEME program identified 20 conserved motifs, which we designated motifs 1 to 20. Most of the *GhMYB* proteins within the same subgroup showed similar motif compositions, while high variance was observed between the different subgroups. This is consistent with previous reports suggesting that MYB family members with similar protein arrangements were classified into the same subfamily [8]. For example, all *GhMYB* proteins in subgroup1 possessed motifs 1, 2, 3, and 12 while all members in subgroup 15 contained motifs 2, 3, 4, 6, 7, and 15 (Additional file 8: Figure S4). In addition, some motifs were specific to a distinctive subgroup, indicative of a particular function of that subgroup. Though the functions of most of the conserved motifs remain to be identified, they are likely to play an important role in the transcriptional regulation of target genes, and may indicate further functional diversification in specific species. Our results suggest that these motifs are evolutionarily conserved and functionally important. This result was similar to Stracke and Dubos who suggested that if MYB genes from the same subgroup share similar protein motifs they probably share similar functions [8, 46].

### Upland cotton MYB family relationships with other plant

**Fig. 2:**
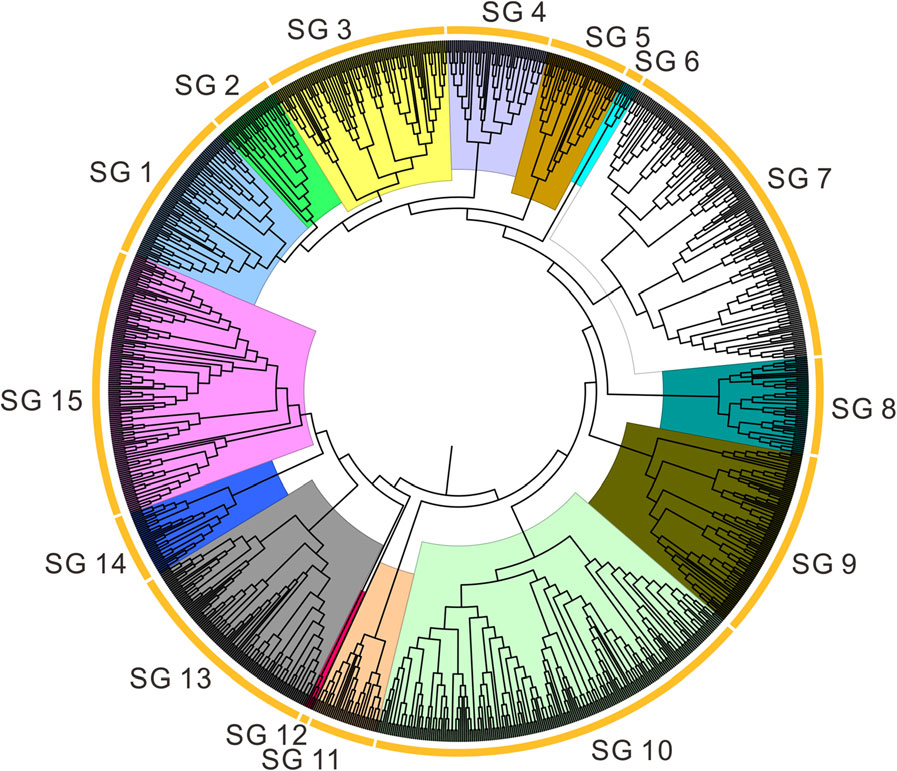
Phylogenetic tree of 492 upland cotton MYB proteins, 197 *Arabidopsis* MYB proteins and cacao 249 MYB proteins. The phylogenetic tree was constructed by MEGA 6.0 using the Neighbor-Joining method. The bootstrap test was performed with 1,000 iterations. The 29 subgroups are shown with different colors

To understand the relationship between the members of the MYB gene family, we constructed an NJ phylogenetic tree of upland cotton, *Arabidopsis,* and cacao MYB proteins. Comparison of protein sequences and phylogenetic tree analysis enabled us to categorize the 524, 197, 141 and 256 MYB genes of upland cotton, *Arabidopsis,* cacao and *Gossypium raimondii,* respectively. We identified 15 subgroups containing 4 to 195 MYB genes (Fig. 2). The bootstrap values for some subgroups of the NJ tree were low as a result of relatively large number of gene sequences that were also found in earlier study [23]. The low bootstrap support for the internal subgroups of those trees was in agreement with phylogenetic analysis of MYBs in other plants [23]. It was likely due to the fact that the MYB domains are comparatively short, and members within a subgroup are highly conserved, with relatively few informative character positions. Most MYB subgroups contained more upland cotton members than *Arabidopsis,* cacao and *Gossyoium raimondii* members except the subgroups 6 as shown in (Additional file 9: Table. S5). Moreover, the classification and identification of the MYB protein sequences was consistent with the previous classification described by Stracke and Dubos [4, 8]. For example, subgroup 1 contained 32 upland cotton, 13 *Arabidopsis*, 9 cacao and 22 *Gossypium raimondii* MYB genes. However, subgroup 14 contained 1 *Arabidopsis*, 3 upland cotton MYB genes. Curiously, some homologs were clustered by species within a subgroup, which referred to that species (Additional file 9: Table. S5). Identification of putative orthologous MYB genes of upland cotton, *Arabidopsis,* cacao and *Gossypium raimondii* was relatively easy because they were clustered in pairs within a subgroup. One hundred forty-nine (90 homologous gene pairs between D5/Dt and 59 between D5/At) orthologous gene pairs were found between upland cotton and *Gossypium raimondii*, while only 1 orthologous was identified between upland cotton and cacao, and 3 orthologous were found between upland cotton and *Arabidopsis*, which might be due to the closer relationships between upland cotton and *G. raimondii.*

In addition, to gain more insights on divergence of the MYB genes after polyploidization, the non-synonymous (Ka) and synonymous (Ks) nucleotide substitutions and their ratio (Ka/Ks) were analyzed for the homologous gene pairs between *G. raimondii* (D5) and upland cotton *G. hirsutum* (Dt). Of the 90 MYB gene pairs D5/Dt homologous, 45 were identical (Ka = Ks = 0 or Ka/Ks ratio = 0), 41 had a Ka/Ks less than 1, suggesting that MYB genes have evolved mainly under the effect of purifying selection. However, only 3 MYB genes had a Ka/Ks ratio more than 1, suggesting that these genes have been evolved by positive selection (Additional file 10: Table S6). This result implying that most of the ancestral MYB genes have been retained in upland cotton *G. hirsutum* after polyploidization.

### Expression profiles of MYB genes in *G. hirsutum*

MYB gene expression was analyzed using RNA-seq data from different stages of cotton fiber development including −1, 1, 3, 5, and 10 DPA. It was noted that 431/524 (82.3 %) MYB genes were expressed in at least one stage of cotton fiber development and the expression 93/524 (17.7 %) MYB genes were not detected by RNA-seq (Additional file 1: Table S1). In addition, of the 431 *GhMYB* genes with detectable expression, 190 exhibited low expression levels during different stages of fiber development (Additional file 6: Figure S2C). According to the phylogenetic tree analysis, the expression of MYB transcription factors can also be divided into 16 subgroups. All genes within subgroups 2, 3 and 5 exhibited low or undetectable expression levels during the five early stages of cotton fiber development. In *Arabidopsis*, this subgroup has been shown to be involved in salt tolerance [47, 48]. *GhMYB*s showed elevated transcript levels in the five cotton fiber developmental periods, suggesting that they might be important for maintenance of metabolic processes and normal cotton fiber development. Most MYB genes in subgroup 1, 4 and subgroups 12 to 15 were highly expressed in the five analyzed stages of fiber development. Many MYB MIXTA-like transcription factors could be involved in the regulation of epidermal cell differentiation in different plant species, including specifying cell shape in petals, vegetative trichome initiation, and branching and seed fiber initiation [26, 27]. Recently, it has been reported that ten MYB (MIXTA-like) genes were highly expressed during early fiber development in *G. hirsutum*. In contrast, only one group of MIXTA-like genes had low expression levels in three natural fiber-less mutants [29]. These mutants provide a means to analyze the roles of MYB transcription factors in the control fiber development in upland cotton. Interestingly, expression of the rice *MYB91* was stimulated by other abiotic stresses and hormone treatment. Moreover, in *Arabidopsis* the *MYB91* gene has been shown to integrate endogenous developmental signals with different environmental conditions [49]. In addition, MYB88 normally maintains fate and developmental progression throughout the stomatal cell lineage [50]. These results indicate that MYB genes can have multiple functions in plant growth and stress responses. Sixty six genes in subgroup 7, CotAD_42675 (MYB2 or GL1), CotAD_18666 (MYB109), CotAD_46807 (MYB109), CotAD_02818 and ect, were highly expressed during the initiation and elongation stages of fiber development, implying that these genes may be involved in a complex network of fiber development. In fact, MYB2 stimulates cotton fiber development [25] and MYB109 is specifically expressed in fiber initiation and elongation stages [24]. Recently, it was found that MYB2 and MYB109 promote normal fiber development in cotton [29]. In this study, 15 MYB-3R genes were identified and clustered into subgroups 10 and 13. The expression of three of the 15 MYB-3R genes could not be detected during cotton fiber development. Therefore, the fact that the MYB-3R family is easily identifiable and characterized could make them suitable targets for genetic engineering approaches aimed at improving cotton fiber development. Previous reports suggest that *GhCPC*, belonging to the MYB-3R subfamily, negatively controls cotton fiber development during the initiation and early elongation stages in mutant cotton [28, 51]. These results are consistent with previous studies which have suggested that MYBs played a crucial role in a wide variety of biological processes including: cell growth, cell cycle control, signal transduction, metabolic and physiological stability, and response to environmental stimuli [37, 52]. Therefore, the MYB family might provide a means to regulate cotton fiber development and offer a path to understanding cell fiber development during the initiation and elongation stages.

### Expression verification of *GhMYB* genes involved in cotton fiber development

**Fig. 3:**
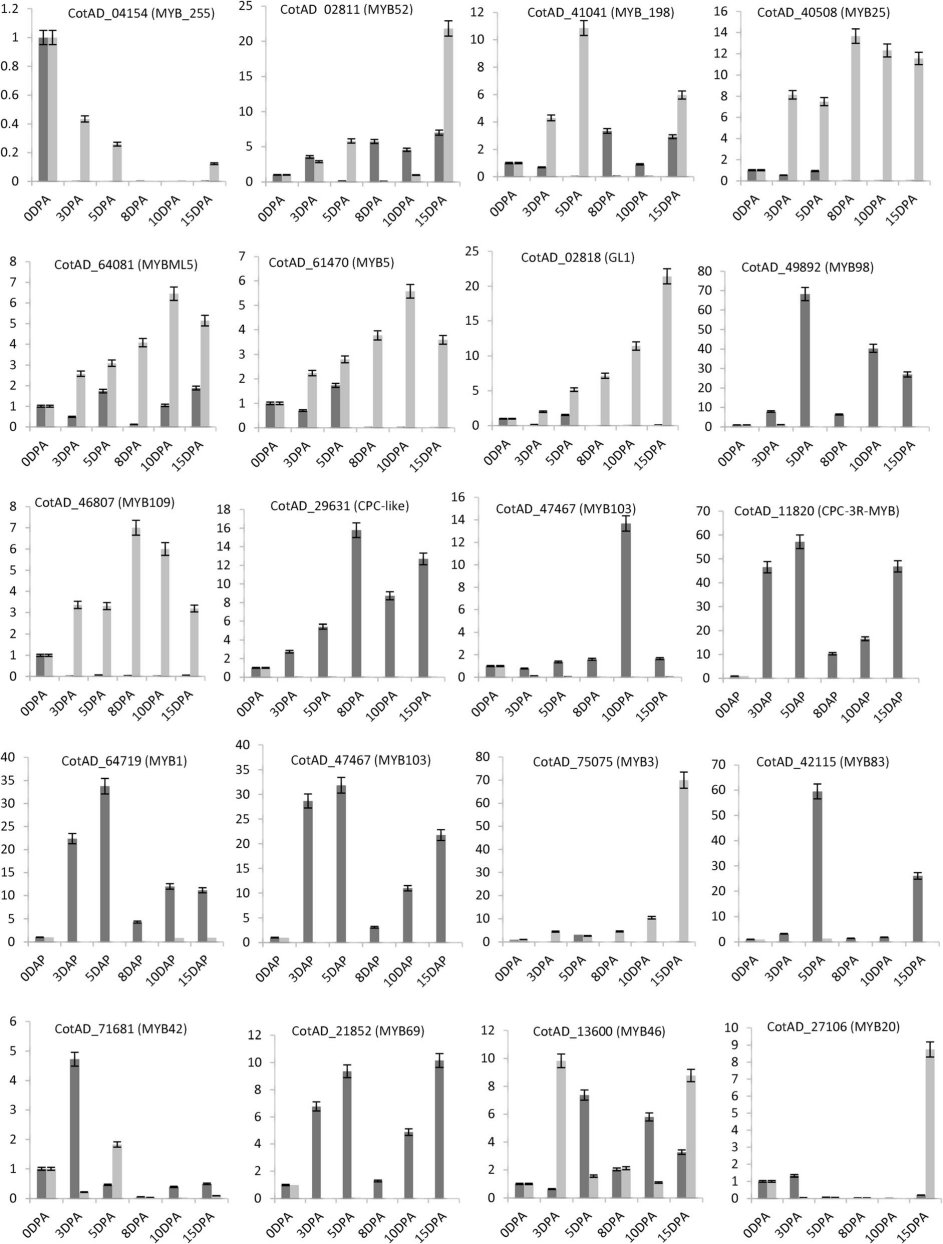
Expression levels of 20 *GhMYB* genes measured by qRT-PCR analysis of Ligon-lintless1 mutant and wild-type at different stages of cotton fiber development. *Black* and *grey* represent the expression levels of *Li1* mutant and wild-type, respectively

MYB transcription factors play roles in many plant specific processes, such as primary and secondary metabolism, cell shape, anthers development, cellular proliferation, differentiation, and stress responses [53, 54]. We randomly selected 20 MYB genes to undergo expression verification using qRT-PCR (Fig. 3). The *GhMYB* genes MYB25, MYB2, MYB109, MYB5, and MYB3 were highly expressed in wild-type *G. hirsutum*, and exhibited lower expression levels in *G. hirsutum* Ligon-lintless1 (*Li*1) mutants after 5DPA. Previously, it found that 8DPA was the critical point for the Ligon-lintless1 mutant [32]. In addition, MYB109 and CotAD_02818 (GL1) promoted cotton fiber development [29]. MYB25 was expressed in ovules (initiation) and fiber development [26]. Our results indicate these genes may play an essential in maintaining normal cotton fiber development. In contrast, some selected *GhMYB* genes such as CotAD_29631 (CPC-like), CotAD_47467 (MYB103), CotAD_11820 (CPC-3R-MYB), CotAD_64719 (MYB1), CotAD_42115 (MYB83), and CotAD_21852 (MYB69) were significantly expressed in the*Ligon-lintless*1 mutant, but not in wild-type. A previous study reported that CPC-3R-MYB negatively controlled cotton fiber development [28], consistent with this the genes that are up-regulated in the Ligon-lintless1 mutant could be responsible for the short fiber phenotype observed. Other groups of genes such as CotAD_04154 (MYB_255), CotAD_02811 (MYB52), CotAD_41041 (MYB_198), CotAD_64081 (MYBML5), CotAD_71681 (MYB42), CotAD_13600 (MYB46), and CotAD_27106 (MYB20) were expressed at different levels in the *Li1* mutant and wild-type which may indicate functional divergence of *GhMYB* genes during cotton fiber development. Previous reports mentioned that MYB genes showed significant expression differences between Ligon-lintless2 and wild-type expression during the later stage of cotton fiber development at 20DPA [55]. Several MYB transcription factors were readjusted by the Ligon-lintless1 mutant at 5 DPA [56], 6 DPA [31], 1 DPA, 3 DPA, and 8 DPA ovules [33]. Overall, it can be seen that our RNA-seq data is consistent with qRT-PCR results. In addition, a comparative expression profile analysis of MYBs in upland cotton revealed that *GhMYB* might have diverse functions at different stages of cell fiber development. Taken together, the RNA-seq and qRT-PCR expression analyses in *G. hirsutum* support the hypothesis that *GhMYB*s are involved in fiber development during different developmental stages, and may have diverse functions in *Arabidopsis* and other species. The functions of most MYBs in higher plants remain unclear, and further investigation is required to elucidate their exact functions. Our results provide a comprehensive understanding of *GhMYB*s and provide the foundation for future functional analyses of MYB genes and their roles in cotton fiber development.

## Conclusions

The MYB gene family is part of the biggest transcription factor family in higher plants and plays an important role in plant growth and development. We undertook a comprehensive genome-wide characterization and expression analysis of the MYB transcription factor family in cotton fiber development. A total of 524 MYB genes were identified and classified into four subfamilies. Based on phylogenetic tree analysis, these MYB transcription factors were classified into 16 subgroups. Proteins within the same subgroup contained very similar gene structures and protein motifs. Additionally, our results revealed that MYB genes were distributed across the entire upland cotton genome. Moreover, RNA-seq data showed that MYB genes play an important role in plants. The expression profiles of 20 genes during cotton fiber development, obtained by qRT-PCR, show that different MYB genes can positively or negatively regulate cotton fiber development. Additionally, other MYB genes are expressed in both mutant and wild-type fiber, further highlighting the diverse functions of MYB proteins in the development of the cotton fiber cell. This study provides strong evidence that *GhMYB* genes play a major role in cotton fiber development and provides a platform for the characterization of interesting MYB genes in the future.

## Additional files

Additional file 1: Table S1. Expression patterns of MYB genes in different stages of cotton fiber development measured by RNA-seq. (XLSX 63 kb) Additional file 2: Table S2. Classification of MYB genes according to MYB repeat domains and protein length. (XLSX 26 kb) Additional file 3: Figure S1. Schematic representation of the general structure of upland cotton R1-MYB, 2R-MYB, 3R-MYB and 4R-MYB domain proteins. (TIF 400 kb) Additional file 4: Table S3. Location of MYB genes in the upland cotton genome. The positive (+) and negative (−) symbols following each gene represent forward and reverse orientations on the chromosome, respectively. (XLSX 45 kb) Additional file 5: Table S4. Synonymous and non-synonymous substitution rates and the estimated times for the tandem and segmental duplication events in *GhMYB* genes. (XLSX 18 kb) Additional file 6: Figure S2. Phylogenetic relationship, gene structure, and gene expression pattern of MYB family in *G. hirsutum* (A) the phylogenetic tree of MYB proteins in upland cotton (B) the exon/intron structure of MYB genes of upland cotton. The blue boxes represent upstream/downstream, yellow boxes represent exons (CDS) and black lines indicate introns. (C) Expression patterns of MYB genes in upland cotton during different stages of cotton fiber development (−1, 1, 3, 5, and 10DPA). The RNA-seq data were analyzed by genesis_v1.7.6.30.09.10-DIGERATI software. Red color represents the MYB genes expressed at higher levels, black represents the genes expressed at lower levels and white color represents the genes that could not be detected by RNA-seq across different stages of fiber development. (TIF 64281 kb) Additional file 7: Figure S3. The neighbor-joining (NJ) method, and minimum evolution methods of MYB family in *G. hirsutum*. (TIF 16793 kb) Additional file 8: Figure S4. Two examples of the conserved protein motifs in the MYB transcription factor family. Each motif is indicated with a specific color. (TIF 1977 kb) Additional file 9: Table S5. The statistical analysis of each group of the NJ phylogenetic tree. (XLSX 13 kb) Additional file 10: Table S6. The Ka/Ks ratios of MYB gene family in cotton (*Gcpb raimodii*/upland cotton). (XLSX 16 kb)
