# Supplementary figures and images for: Genome-wide characterization and expression analysis of MYB transcription factors in Gossypium hirsutum
Source: BMC Genet. 2016 Sep 9;17(1):129. doi: 10.1186/s12863-016-0436-8 (PMC5017022; doi:10.1186/s12863-016-0436-8)

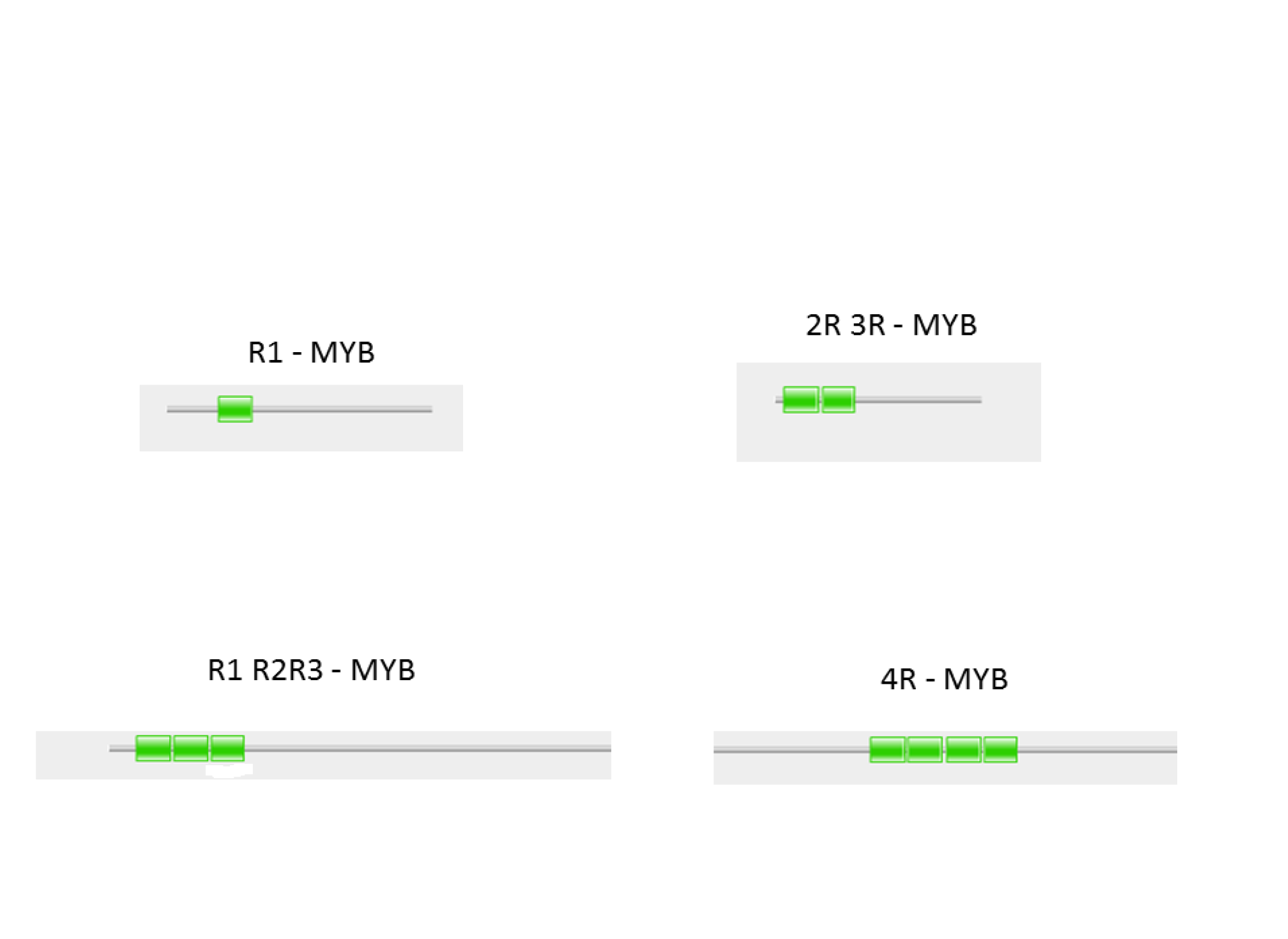

Supplement: Additional file 3: Figure S1. — Schematic representation of the general structure of upland cotton R1-MYB, 2R-MYB, 3R-MYB and 4R-MYB domain proteins. (TIF 400 kb) [file 12863_2016_436_MOESM3_ESM.tif]

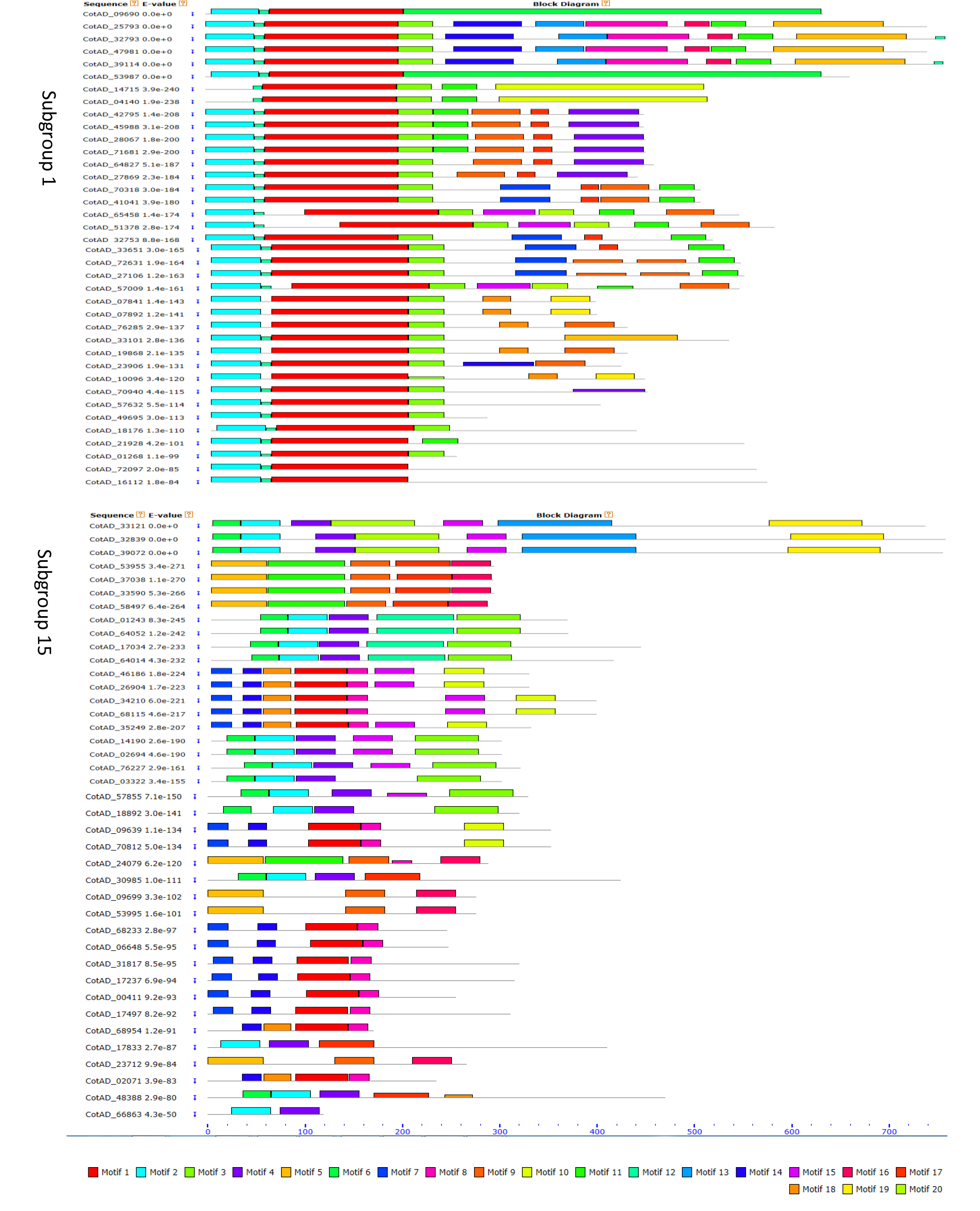

Supplement: Additional file 8: Figure S4. — Two examples of the conserved protein motifs in the MYB transcription factor family. Each motif is indicated with a specific color. (TIF 1977 kb) [file 12863_2016_436_MOESM8_ESM.tif]
